# Breastfeeding among contemporary Australian populations and 2025 national targets: a scoping review of current data and implications for policy and practice

**DOI:** 10.1186/s13006-026-00826-9

**Published:** 2026-04-14

**Authors:** Maria Oliveri, Jeanine Young, Kym M. Rae, Vicki Clifton

**Affiliations:** 1 Faculty of Health, Medicine and Behavioural Sciences, The University of Queensland, Brisbane, Queensland Australia; 2 Pregnancy and Development Group, Indigenous Health Research Group, Mater Research Institute, South Brisbane, Queensland Australia; 3 School of Health, The University of the Sunshine Coast, Petrie, Queensland Australia; 4 Queensland Kids Partnership Intergenerational Stewardship Table, Queensland Health, Brisbane, Queensland Australia

**Keywords:** Breast feeding, Australia, Australian Aboriginal and Torres Strait Islander Peoples, Health policy, Health equity, Perinatal care, Public health

## Abstract

**Background:**

Since the 2010 Australian National Infant Feeding Survey, reliable breastfeeding data of comparable scale has been scarce, with annual national data remaining small in sample size and non-representative of diverse Australian populations. National 2025 breastfeeding targets, aligned with global nutrition targets, have not been evaluated. National breastfeeding data largely ceases upon immediate postpartum hospital discharge, resulting in the inability to monitor breastfeeding beyond this timepoint. Consequentially, maternal and child health priorities aimed to reduce health inequities among ethnically diverse and Indigenous Australians, also prove difficult to determine. Accountability towards achieving targets, through ongoing monitoring and evaluation of progress is difficult to establish, as breastfeeding remains excluded from national health performance frameworks and mandatory reporting systems.

**Methods:**

A scoping review was conducted in accordance with Preferred Reporting Items for Systematic Reviews and Meta-Analyses (PRISMA-ScR) to identify peer-reviewed publications reporting on breastfeeding practices among Australian populations, since 2010. Identifying recent literature reflecting breastfeeding exclusivity, prevalence, durations, enablers and/or barriers in contemporary and diverse, nationally representative Australian populations, allowed for critical appraisal of Australian breastfeeding surveillance, in comparison to nationally reported breastfeeding data.

**Results:**

Inconsistent definitions and reporting of breastfeeding practices resulted in breastfeeding intention, exclusivity and durations varying greatly between population sub-groups identified within the included studies. Durations of 0–6 months accounted for 68% of papers, with 29% of these reporting breastfeeding status at hospital discharge only. Marginalised ethnically diverse and Indigenous Australian populations face unique barriers to achieving breastfeeding targets, with implications to policy and practice baring special consideration.

**Conclusion:**

Since 2010, Australian breastfeeding rates reported have remained unreliable and non-representative of diverse and marginalised populations. A national, standardised breastfeeding data collection framework, allows for accurate and ongoing documentation of infant feeding from birth, through to toddler years, to effectively evaluate national targets or equity progress. Including breastfeeding as a mandatory national health indicator with standardised national reporting systems, ensures policy accountability to monitor outcomes and guide resource allocation to support targeted interventions for marginalised populations. Cohesive national policy and legislation to protect, promote and support breastfeeding, reduces structural and societal barriers faced in achieving equitable and optimal breastfeeding outcomes.

**Supplementary information:**

The online version contains supplementary material available at 10.1186/s13006-026-00826-9.

## Background

Human breastmilk is a biologically complete food source, providing all essential nutrients for infants during the first six months of life [1]. The benefits of breastfeeding are well established [2, 3], underpinning the World Health Organization’s (WHO) recommendation for exclusive breastfeeding (EBF) to six months, with continued breastfeeding to two years and beyond [1]. EBF refers to breastmilk as the sole source of nutrition; any intake of other fluids or solids classifies the infant as non-EBF [2].

For infants, breastfeeding reduces risk of respiratory and gastrointestinal infections, asthma, eczema, allergies, obesity and chronic diseases in adulthood [2, 3]. Its bioavailable iron supports healthy neurodevelopment and helps prevent iron deficiency anaemia, which is linked to poor cognitive, behavioural, and mental health outcomes [3–5]. Breastfed children also demonstrate improved socio-emotional wellbeing and fewer behavioural disorders [5, 6]. According to the 2023 Australian Institute of Health and Welfare (AIHW), mental health conditions - including anxiety, depression; along with neurodevelopmental disorders, such as autism spectrum disorder (ASD), and behavioural issues, are among the leading causes of disease burden in Australian children, second only to asthma [7].

For mothers, breastfeeding lowers future risk of developing gestational diabetes, type 2 diabetes, reproductive cancers, metabolic syndrome and cardiovascular disease [8, 9]; the leading cause of death for women globally, with particular relevance for high risk groups [10, 11].

Breastfeeding is especially important for marginalised Australian populations, including culturally and linguistically diverse (CALD) and Aboriginal and Torres Strait Islander populations (hereafter the term Indigenous Australians will respectfully be used), who have lower overall breastfeeding rates and higher rates of chronic disease [7, 10]. CALD refers to individuals born outside Australia, or those for whom English is not their primary language [10]. These groups face systemic inequities, language barriers and cultural disconnection, contributing to poorer breastfeeding outcomes and long-term health [7, 10].

The economic cost of suboptimal breastfeeding is significant. The AIWH estimated the annual cost to the Australian health system of A$120 million, due to preventable childhood illnesses, while infant formula manufacturing produces twice the carbon emissions of breastmilk [7]. Rising chronic disease rates are driving increased healthcare spending, with curative care comprising 70% of total costs [10, 12]. Tools like the Mothers’ Milk Tool aim to quantify breastmilk’s economic value by including it in Gross Domestic Product (GDP) calculations [13, 14]. Over a decade ago, Smith (2013) estimated Australian breastmilk production at A$3 billion annually [15].

Social determinants of health, such as education, income, employment, culture, rurality, colonisation, and access to health services all influence breastfeeding behaviours [10, 16]. To address health services influence in breastfeeding initiation and continuation [17–19], the WHO and United Nations Children’s Fund (UNICEF) introduced the “10 Steps to Successful Breastfeeding” under the Baby Friendly Health Initiative (BFHI) [1]. This global standard promotes supportive hospital policies and care practices. In Australia, only 25% of hospitals meet full BFHI accreditation, subjecting mothers and infants to inadequate breastfeeding support during the critical early postpartum period [20].

The Australian National Breastfeeding Strategy: 2019 and beyond, set a goal for 50% of Australian infants to be EBF through to 6 months of age by 2025 [21]; aligning with the WHO targets. Australia’s National Health and Medical Research Council (NHMRC) recommends breastfeeding “until around 12 months” [22], reinforcing cultural norms that fall short of the WHO’s recommendation of two years and beyond [2]. In addition to cohesive national breastfeeding statements, four key policy strategies are essential to protect and support breastfeeding include: adequate paid parental leave, workplace protections, regulation of formula marketing, and economic and climate policy integration [23–25].

Six months of postnatal paid parental leave can reduce gender inequities and support sustained breastfeeding by easing pressure to return to work - a major barrier to continued breastfeeding [25]. Workplace legislation must mandate lactation breaks and protections for breastfeeding employees to support continued breastfeeding upon return to work [19]. In Australia, breastfeeding is legally protected under the federal *Sex Discrimination Act 1984*, which prohibits direct and indirect discrimination against breastfeeding women across employment and public settings, alongside complementary state and territory laws [26]. The WHO/UNICEF International Code of Marketing of Breastmilk Substitutes (The Code, WHA) calls for strict, enforceable regulation of artificial milk marketing [27]. However Australia’s outdated “Marketing of Artificial Infant Formula” (MAIF) Agreement (1992), currently under review, has been criticised for favouring industry interests over public health [28, 29]. New mandatory legislation is expected within two years (26), to protect parents from misleading marketing and strengthen breastfeeding promotion [29, 30].

Australia faces major challenges in breastfeeding data collection and quality, undermining efforts to improve maternal and child outcomes, including “Closing the Gap 2032” targets to reduce health disparities for Indigenous Australians [31]. Despite breastfeeding’s well-established role in preventing chronic disease, comprehensive national data is lacking. The most reliable dataset remains the 2010 Australian National Infant Feeding Survey (ANIFS), which found only 39% of infants were EBF to 3 months, and 15% to 5 months [32]. However, despite 27.6% of Australians being born overseas and 7.5% speaking another language other than English at home, this survey excluded many CALD families due to its English-only format [33].

Since 2010, limited data has been collected. The Australian Bureau of Statistics (ABS) offers only biannual updates based on small samples (1000–1500 children aged 0–4 years), excluding remote regions, discrete Indigenous communities and people in temporary housing [7]; groups with poorer health outcomes and lower breastfeeding rates (4).

To improve representation, the ABS introduced targeted national health surveys for Indigenous Australians, with updated breastfeeding data collected in 2018–2019 and 2022–2023 [10]. While overall rates remain lower than the general population, remote Indigenous communities show higher EBF rates: 41% at 6 months compared to just 12% of Indigenous infants in metropolitan areas [11]. The ANIFS reported EBF at 5 months was 27% for non-Indigenous infants, and 11% for Indigenous infants [32]. CALD infants also face lower breastfeeding rates, although exact rates were not reported [34].

Australia’s lack of routine, nationally representative breastfeeding data hinders future policy development and current policy accountability, with absent monitoring systems hindering progress toward bridging significant lifelong health disparities experienced by marginalised populations. This review addresses these gaps by examining recent data to inform stronger public health policy and improve maternal and child health outcomes across all communities.

## Methodology and methods

### Design

A scoping narrative review was conducted following the PRISMA-ScR guidelines [35] and Joanna Briggs Institute (JBI) methodology [36] to map evidence, clarify concepts, and identify gaps-particularly relevant to fragmented breastfeeding data [37]. The research question posed was: “What are the current reported breastfeeding rates in Australian populations since 2010, in comparison to ABS national breastfeeding data, and what enablers or barriers of optimal breastfeeding have been identified within the literature?”

The specific objectives of this review were to explore peer-reviewed studies reporting breastfeeding rates within contemporary Australian populations since the 2010 ANIFS to: a) identify peer-reviewed publications addressing breastfeeding exclusivity, prevalence and duration in Australian populations, including marginalised CALD and Indigenous Australian populations, b) evaluate methodologies used to assess rigor in comparison to national ABS and AIHW data, and c) identify enablers and/or barriers.

A scoping review was undertaken to synthesise and critically appraise Australia’s breastfeeding surveillance by mapping recent literature reporting breastfeeding outcomes against national data. This approach was chosen to enable inclusion of diverse study designs and populations, particularly marginalised groups, and allow integration of reported barriers and enablers alongside outcome data. The evidence-base was used to identify surveillance gaps and policy implications for achieving national targets. A systematic review was not appropriate due to substantial heterogeneity in breastfeeding definitions, outcomes, data sources and surveillance purposes, which precluded meaningful comparison or pooling of results. State-based datasets were excluded as they reported rates only, duplicated national findings, and omitted small-volume hospitals, thereby underrepresenting populations experiencing persistent inequities.

### Eligibility criteria

Details of included studies and eligibility criteria can be found in Table 1 and supplementary files.

**Table 1:** Study eligibility: inclusion and exclusion criteria

Inclusion	Exclusion
Year of publication: 2011- April 2025	Article not available in English
Peer-reviewed publication	Not peer-reviewed
Measuring breastfeeding (initiation, duration or feeding method)	Retrospective samples occurred before 2010
Retrospective Samples recruited after 2010	Analysing 2010 ANIFS Data
All study types/designs (qualitative, quantitative, cohort (prospective and retrospective), cross-sectional, mixed methods)	Australian populations included, but not discussed separately (eg: Australia and New Zealand)
Measuring breastfeeding within 0–24 month timeframes	State-based perinatal breastfeeding data
Australian populations, living in Australia	
Article available in English	

### Search strategy: sources and search

Databases searched in April 2025 included: CINAHL, Medline, PubMed, Scopus, Cochrane, Embase, using identified keywords and index terms, together with a secondary hand search of reference lists and limited grey literature search. Search terms and strategy (in supplementary files) were developed and agreed on by the research team and a university librarian. Final search results were exported into Endnote.; duplicates were excluded.

**Fig. 1:**
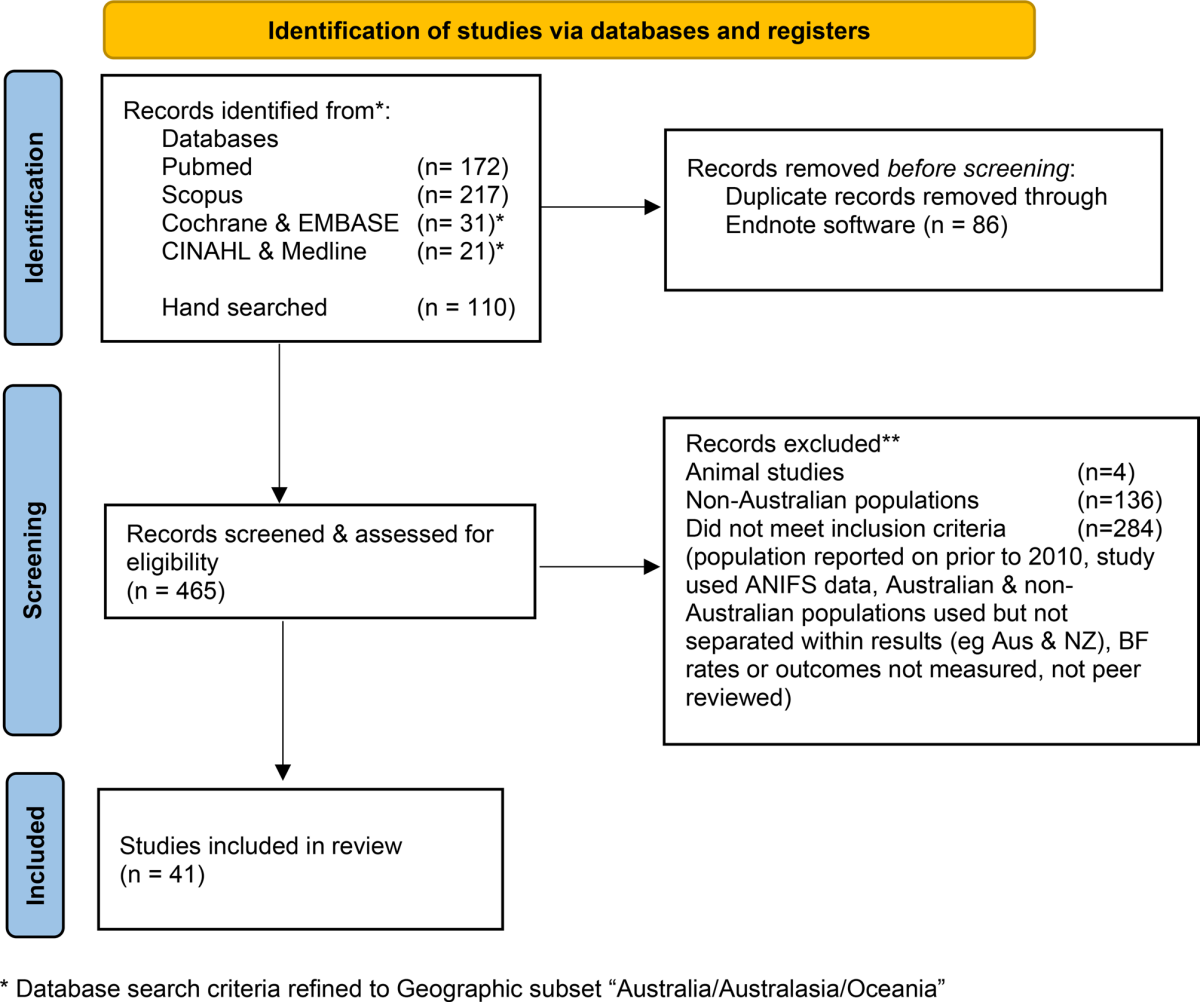
PRISMA flowchart of identification of studies for scoping review

The PRISMA flowchart (Fig. 1) summarises the process of selecting studies for inclusion in this review.

### Data extraction and analysis

One reviewer (MO) selected studies and extracted data based on the primary research question and eligibility criteria, in consultation with the co-author team. Content from eligible articles were extracted from Endnote. (Clarivate Analytics, PA, US) to Excel and data summarised categorically, using JBI data extraction templates, to ensure information retrieved answered the review aim and research question [35]. The research team discussed studies that were borderline in meeting inclusion criteria and reached a consensus on inclusion and exclusion. Included papers were analysed using JBI Risk of Bias (RoB) tools [38].

## Results

### Study selection

The initial search identified 551 peer-reviewed articles. After removing duplicates (*n* = 86), 465 titles and abstracts were screened against eligibility criteria. Of these, 424 were excluded due to non-Australian or animal sampling (*n* = 4) or failure to meet inclusion criteria (*n* = 284). Two studies with data collected in 2009–2014 [39] and 2009–2013 [40] were included, as most data were post-2010 ANIFS. In total, 41 studies met established inclusion criteria and were reviewed (Fig. 1); study details and sample sizes are in Supplementary Files. Table 2 summarises extracted data. Data are categorised by population sub-groups (Indigenous, CALD, general Australian) and ordered by breastfeeding duration within each subgroup.

**Table 2:** Summary of eligible studies categorised by population sub-groups

Authors	Study Period	Aims	Design/ Method	Population Sub-groups	Sample size & Location (city, region/state)	Findings: Intention	Findings: Initiation	Findings: Duration (order)	Total BF duration assessed
**INDIGENOUS AUSTRALIANS**									
Kildea, et al. [41]	2013–2019	Evaluation of the Birthing in Our Community (BiOC) First Nations model of care & maternal and infant health outcomes vs standard care including; antenatal visit attendance, smoking in pregnancy, preterm birth (<37 weeks) & EBF at hospital discharge.	Prospective, non-randomised, interventional trial	First Nations infants in BiOC vs standard care models	(*n* = 1,422) Brisbane, Metropolitan QLD	90% intention to BF in both groups.		EBF on discharge: 73.9% BiOC mothers 67.6% standard care	**BF at discharge**
Springall, et al. [42]	2017–2020	Evaluation of BF outcomes of First Nations infants, in a culturally specific continuity of midwifery care model including; reasons for stopping and factors associated with BF.	Prospective longitudinal cohort study	Mothers birthing in First Nations continuity of care	(*n* = 211) 3 sites for Baggarrook Yurrongi study in Melbourne, Metropolitan VIC	Intention to: EBF 77%. BF & AF 18%. BF 3 months or more 54%. BF 6 months or more 48%	96%	3 months: 71% any BF, (of these 48% EBF)	**3 months**
Austin, et al. [39]	2009–2014	To Improve engagement of Aboriginal families in Maternal Child Health (MCH) services for 0–6 year olds	Cross-sectional study	Aboriginal families	(*n* = 146) Glenelg Shire, Metropolitan VIC		Increased from 67% to 84%	Any BF to 6 months increased from 17% to 64%	**6 months**
Longmore, et al. [43]	2011–2017	Evaluation of association of hyperglycaemia (in GDM and T2DM), with BF outcomes in Indigenous and non-Indigenous mothers of the Pregnancy And Neonatal Diabetes Outcomes in Remote Australia (PANDORA) cohort	Retrospective longitudinal cohort study	Mothers with GDM & T2DM including Indigenous & non-Indigenous populations	(*n* = 1,050) Remote NT			EBF at discharge: T2DM Indigenous mothers, 52% T2DM non-Indigenous mothers, 29% Predominant BF 6 weeks: Indigenous 82% vs non-Indigenous 66% Indigenous GDM 81% vs 57%, non-Indigenous GDM, T2DM Indigenous 61% vs T2DM non-Indigenous 33%. Predominant BF 6 months: Indigenous 71% vs non-Indigenous 60%. Indigenous GDM 68% vs non-Indigenous GDM 46%. T2DM Indigenous 58% vs T2DM non-Indigenous 40%	
Brown, et al. [44]	2011–2013	Comparison of initiation and duration of BF of Aboriginal infants in urban, rural and remote regions	Cross-sectional, population-based study	Aboriginal Infants. Culturally specific models of care.	(*n* = 344) State-wide, SA Urban, rural, remote regions		85%	EBF > 6 weeks: 60% of infants in metro areas, compared with 79% of infants in remote areas Any BF at 12 weeks: 60% in urban areas 79% in remote areas 54% Any BF at 12 weeks: 67% in regional standard care in vs 61% in regional Aboriginal model. Any BF 12 weeks: 36% in standard metro AN care vs 49% metro Aboriginal model.	**4–12 months**
Leonard, et al. [45]	2010–2012	Description of first foods of Aboriginal and Torres Strait Islander infants and young children in a nutrition & anaemia prevention study	Cross sectional survey	Aboriginal and Torres Strait Islander infants & children aged 6–24 months	(*n* = 227) Remote, northern WA, NT & QLD			In 24 hours pre-survey, receiving any BM: 67.4% of all children 80% of 6–12 month olds 52% of 12–24 month olds	**24 months**
Onifade, et al. [46]	2010–2018	Evaluation of BF intentions and practices of mothers of First Nations babies in the Gomeroi Gaaynggal cohort	Prospective longitudinal cohort study	Aboriginal and Torres Strait Islander infants	(*n* = 71–246) Tamworth, rural NSW	BF intention 72.8%	83.9% received any BM at birth	Any BF: 30% at 3 months. 8.9% at 4–6 months, 5.1% >12 months, 0% at 24 months.	
Tonkin, et al. [47]	2017–2018	Evaluation of dietary intake of children 6–36 months in a remote Aboriginal community including understanding milk feeding & diet when solids introduced, compared to national recommendations.	Cross-sectional survey	Aboriginal mothers & children	(*n* = 40) Aboriginal communities, Remote NT			Any BF rates 85% overall. 67% of >2 years olds BF.	**3 years**
Ashman, et al. [48]	2012–2016	Evaluation of PN dietary intakes, anthropometric outcomes of Indigenous mothers and children	Cross-sectional evaluation of cohort study	Indigenous mothers & children	(*n* = 73) Tamworth region, rural NSW		85.9%	Any BF median 1.4 months. Any BF at: 3 months 24% 6 months 29% 12 months 6.7% 12 months-5 years 6.7%	**5 years**
**CALD AUSTRALIANS**									
Dahlen, et al. [49]	2012–2013	Comparing obstetric and psychosocial risk profiles and maternal and neonatal outcomes between Australian-born and non-Australian born women	Cohort study	Australian born vs non-Australian born mothers (CALD) EPDS.	(*n* = 3,092) Sydney, Metropolitan NSW			79% BF with EPDS < 12 69% BF with EPDS > 13 (high)	**BF at discharge**
De Mare, et al. [50]	2016–2018	Comparing normal birth (NB) vs Caesarean birth (CB), and feeding regimes of exclusive breast milk associated with early postnatal readmission	Retrospective cohort study	CB vs NB. Ethnicity: Aus/NZ/Pacific, ATSI, Asian, middle-eastern (CALD),EPDS	(*n* = 5,520) Epping, Metropolitan VIC			62.8% EBF at discharge, Similar BF rates between NB vs CB groups, Em CB lower BF rates	
Melov, et al. [51]	2018–2021	Assessing the impact of the COVID-19 pandemic on perinatal outcomes in an Australian high migrant and low COVID-19 prevalent population to identify if health service changes impact obstetric and perinatal outcomes.	Retrospective cohort	Pre-COVID vs 1st year of COVID. non-Australian born (66%) CALD population	(*n* = 34,103) Western Sydney, Metropolitan NSW			1st year of COVID 15% less EBF at discharge than pre-COVID. EBF at discharge: 65.8% pre-COVID vs 62% during COVID	
Ogbo, et al. [52]	2014	Assessing the prevalence and determinants of cessation of exclusive breastfeeding (EBF) in the early postnatal period in a CALD population in Sydney.	Retrospective cohort	CALD, over 40% overseas-born	(*n* = 17,564) Sydney, Metropolitan NSW	BF 92%		EBF: at delivery 90%, at hospital discharge 89% between 1 and 4 weeks 62% AF only 16%	**4 weeks**
Ogbo, et al. [53]	2014–2016	Investigating the determinants of EBF cessation in the early postnatal period, among CALD mothers, by evaluating BF intention, EBF at birth, discharge & 1–4 weeks PN.	Retrospective cohort	CALD mothers	(*n* = 25,407) Sydney, Metropolitan NSW	BF 94.7%	91%	EBF at discharge 93.0%, EBF in 1–4 weeks PN 91.4%.	
Keir, et al. [54]	2018–2020	Assessing early BF practices on length of stay & maternal and infant factors associated with BF duration. Focus on feeding from birth to 6 months corrected age (CA) in infants born late preterm (34+0–36+6) with mothers intending to BF.	Prospective cohort study	late preterm infants (34+0–36+6), CALD	(*n* = 270) Metropolitan, SA	84% planned to initiate. 64% intended to BF for at least 6 months	97% received BM.	feeding in hospital: 78% received BM as their first feed 17% received BM only 83% received any AF Overall late preterm EBF: 74% at discharge 41% at 6 weeks 35% at 3 months 29% at 6 months. EBF & any BF combined: 72% at 6 weeks 64% at 3 months 53% at 6 months	
Kuswara, et al. [55]	2018	Evaluating infant feeding patterns in the first 12 months, and factors associated with EBF and AF introduction among Chinese Australian mothers. Chinese ethnicity included the mother or her parents born in mainland China, Taiwan or Hong Kong.	Cross sectional survey	Chinese Australians (CALD)	(*n* = 289) Australia-wide (online)	EBF intention 61%	93% initiated BF	EBF at: 1 month 44% 4 months 33% 6 months 18% Any AF at: Birth 7% 1 month 55% 6 months 63% Any BF at: 6 months 81% 12 months 50% 24 months 21%.	**24 months**
**GENERAL AUSTRALIAN POPULATIONS**									
Bish, et al. [56]	2010–2017	Assessing BF initiation from routinely collected hospital data, in a large Australian regional hospital, to assess associations between obesity and BF.	Retrospective, Population-based cohort	Obese (BMI > 30), non-obese	(*n* = 10,234) Metropolitan VIC		78.9% for maternal BMI > 30. vs 87.1% for maternal BMI < 30	15.4% AF 62.5% EBF 22% Any BF (direct BF/expressed BM and AF)	**BF at discharge**
Cummins, et al. [57]	2018–2021	Comparing pre-term birth rates and BF rates in women with a mental health history, in midwife group care and standard models of maternity care.	Retrospective cohort study	Perinatal mental health conditions and models of care	(*n* = 3,028) Newcastle, Metropolitan NSW			In MGP: 91% EBF Standard care: 70% EBF	
Flood, et al. [40]	2009–2013	Assessing the relationship between PPH > 500 ml/severe PPH > 1500 ml and BF outcomes	Retrospective, population-based cohort study	PPH vs Severe PPH	(*n* = 339,854) State-wide, VIC		BF initiation 94.9%. 79% had first BF at breast (not EBM).	AF given 24% EBL < 500 ml, 34% >500 ml, 44% EBL > 1500. PPH & severe PPH less likely EBF at discharge. From PPH initiated BF, 79% pre-discharge feed BF.	
Jarrett, et al. [58]	2018	Assessing the maternal and neonatal factors associated with neonates readmitted to hospital within 28 days of birth	Case controlled, cross-sectional, retrospective cohort study	neonatal readmission vs control	(*n* = 251) Sydney, Metropolitan NSW			69% of readmissions EBF at discharge (68% in control). 20% BF & AF 10% AF	
Jones, et al. [59]	2018–2020	Assessing the effect of a short-stay (<72 hours) admission to the neonatal unit vs no admission, on BF outcomes at hospital discharge.	Retrospective cohort study	admission to a neonatal unit vs no admission	(*n* = 1,000) Western Sydney, Metropolitan NSW			EBF at discharge: neonatal unit admitted 64% vs no admission 84.6%	
Meedya, et al. [17]	2018–2019	Evaluating infant feeding data in Australian hospitals and compare outcomes between BFHI and non-BFHI accredited hospitals, and between public and private hospitals.	Retrospective cohort study	NSW vs VIC, Public vs Private, BFHI vs non-BFHI	(*n* = 200,000) State-wide NSW & VIC (*n* = 125 hospitals, >200 births per annum)			NSW: Full BF at discharge 74% in Public vs 66% in Private Hospitals. NSW: Partial BF 24% in Private vs 12% public. VIC: EBF at discharge 75% in Public vs 69% in Private VIC Hospitals. NSW: Public non-BFHI: 10.4% formula only vs Public BFHI 5.4%. Private non-BFHI 67.7% EBF vs Public non-NFHI 74.5%. VIC: Private, 42% BF babies had AF in Hospital, vs Public 22%.	
Mollart, et al. [60]	2022–2023	Evaluating perinatal outcomes between Midwifery Antenatal and Postnatal Service (MAPS), known midwife perinatal care & Midwifery Group Practice (MGP) models of care	Retrospective cohort study	MAPS vs MGP care.	(*n* = 1,303) Outer Newcastle, Rural NSW			EBF at discharge: MGP 83.6% MAPS 74.8%	
Perrella, et al. [61]	2022	Exploring the facilitators and barriers to establishing BF by Australian women birthing via Caesarean section	Mixed-methods study	non-EL CS vs EL CS. Primiparous vs multiparous 86.3% Aus/British, 51.9% BMI > 25	(*n* = 961) Perth, Metropolitan WA	BF duration intent mean = 12.5 months	70.2% initiated BF within 1 hour	Any BF in Hosp: Lowest for Non-EL CS (75%) and primiparous (74%).	
Sweet, et al. [62]	2009–2019	Evaluating the maternal and neonatal outcomes of 10 years of publicly funded homebirths in Australia.	Retrospective cohort study	Public home birth vs Public hospital birth	(*n* = 774) Melbourne, Metropolitan VIC		Hospital birth 98% Home birth 99%	EBF on discharge: Home births 99.8% vs hospital 98.5% of eligible participants	
Holton, et al. [63]	2020–2021	Evaluating midwives perspectives on barriers and enablers to BF for women with high BMI (>25) vs normal BMI (<25). survey in pregnancy & PN about BF intentions, barriers, enablers and support preferences.	Mixed-methods: quantitative (surveys) qualitative (focus group) study	High BMI **(>25)** vs Normal BMI	(*n* = 80) Melbourne, Metropolitan VIC	Intention Any BF 94%. EBF 75.8%. EBF to 6 months 65%	Initiation: EBF at discharge 61%	EBF 6–8 weeks 53% No significant differences between High vs normal BMI. PN, only 34% intended to EBF to 6 months EBF from all participants: in hospital 68%, at discharge 61%6–8 weeks postpartum 53%	**6–8 weeks**
Cole, et al. [64]	2017	Investigating factors associated with BF duration and cessation, to support & advance optimal BF practices, to reduce infant deaths.	Cross-sectional study	any BF vs non-BF. Bed sharing vs non bed-sharing. Pacifier use vs no pacifier	(*n* = 3,321) State-wide, QLD			at 8 weeks: no BF 17.2% any BF 82.8%	**8 weeks**
Reynolds, et al. [65]	2019–2020	Investigating intention to BF and subsequent BF practices, in women who birthed in the last 8–21 weeks; to describe reasons for BF cessation prior to the infant being 5 months of age; examining associations between maternal, pregnancy & infant characteristics.	Cross-sectional study	intention to BF vs actual BF outcomes	(*n* = 536) Australia-wide (online)	94%	95%	57% EBF at time of survey (baby 8–21 weeks old). 2–3 month olds: 60% EBF, 25–30% not BF, 10–14% BF & AF. 4 month olds: 54% EBF, 27% not BF, 12% BF & AF, 7% BF & solids 7%. 5 month olds: 29% EBF 29%, 38% not BF, 19% BF & AF, 14% BF & solids	**8–12 weeks**
Perrella, et al. [66]	2015–2016	Describing BF characteristics of mother–preterm infants discharged receiving any BM (BF or EBM) up to 12 weeks corrected gestational age (CGA).	Prospective longitudinal cohort study	pre-term infants receiving any BM on discharge	(*n* = 49) Perth, Metropolitan WA			At 12 weeks CGA: 59% receiving any BM (of these, 31% EBF at breast, 47% exclusively BM fed).	**3 months**
Bailey, et al. [67]	2014–2015	Exploring the association between infant sleeping location, EBF, BF duration and predictors of greater BF duration in mothers with strong BF outcomes.	Cross sectional survey	ABA-trained volunteer BF counsellors	(*n* = 174) Australia-wide (online)			EBF to 6 months: 61% infants who co-slept, 72% EBF; infants who slept in another room: 51% EBF.	**6 months**
Fan, et al. [68]	2018–2020	Evaluating lactation consultant telephone support & BF rates at 1, 3 & 6 months postpartum, for mothers at risk early BF cessation.	Prospective cohort observational study	Mothers at risk of early BF cessation. Intervention vs control groups.	(*n* = 765) Epping, Metropolitan VIC			Intervention: 1 month 70% Primips EBF. 73% of intervention group EBF at 1 month, 52% at 6 months. Control: 1 month 45% Primips EBF. 55% of controls EBF at 1 month and 36% EBF at 6 months.	
Forster, et al. [69]	2013–2015	Evaluating whether regular postnatal telephone-based peer support increases the proportion of infants BF at six months	Unblinded randomised control trial	Primiparous receiving BF peer support vs control	(*n* = 1,152) multi-centre, Metropolitan VIC			Intervention at 6 months: 75% any BF. 54% EBF Control at 6 months: 69% any BF. 48% EBF.	
Leow, et al. [70]	2014–2015	Examining infant feeding practices & education levels of parents in 3 sites: Campbelltown (South Western Sydney), Australia, Singapore and Ho Chi Minh City, Vietnam.	Cross sectional survey	Surveys in English (Australia & Singapore) and Vietnamese (Vietnam)	(*n* = 108) South-west Sydney, Metropolitan NSW			Sydney population EBF: At discharge 61%, 4 months 39.8% 5 months 32.4% 6 months 21.3%	
McLachlan, et al. [71]	2012–2013	Assessing the effectiveness of 2 community-based interventions in 10 local government areas of Victoria, with low BF initiation rates. Evaluating if BF duration increased of ‘any’ BF of infants at 3, 4 and 6 months.	3-arm cluster randomised trial	Groups: 1) Usual care 2) home visit 3) home visit & drop-in (health clinic)	(*n* = 6,916) State-wide, VIC			Any BF at (across all 3 intervention groups) 3 months 58% − 66% 4 months 53.9% − 63.3% 6 months: 44.5% − 53.6%	
Moss, et al. [72]	2016–2017	Establishing maternal demographics & reasons for not EBF to 6 months, from the Mothers and Their Children’s Health Study.	Retrospective cohort study	EBF vs non-EBF to 6 months	(*n* = 5,340) Australia-wide			34.4% EBF to 6 months. Five non-EBF practices identified: never BF (3.9%), BF < 6 months (20.8%), BF to 6 months with AF (6.8%), BF & solids (24.5%), AF and solids (9.7%).	
Newby, et al. [73]	2010–2012	Examining the relationship between maternal pre-gravid (BMI), BF duration, AN BF confidence, intention, social comfort in primiparous Australians.	Longitudinal cohort study	BMI < 30 vs BMI > 30	(*n* = 462) Brisbane, Metropolitan QLD	AN BF intention: BMI < 30, 96.8%, BMI > 30, 90%. AN BF intend duration: BMI < 30 12.5 months, BMI > 30, 11.5 months	97%	At 12 months: 54% of BMI 18.5–24.9 mothers still BF 40% of BMI 25–30 mothers still BF 20% of BMI > 30 still BF	**12 months/** **1 year**
Wen, et al. [74]	2017–2018	Assessing the effectiveness of telephone or SMS support in improving infant feeding practices, tummy time and reduced screen time.	Parallel randomised control trial (RCT)	Telephone support or SMS support vs control	(*n* = 1,155) Sydney, Metropolitan NSW			Across 3 groups: EBF at 6 months 3–6%. Any BF at 6 months 71–73%. BF at 12 months higher (49%) in both intervention arms vs control (44%).	
Bond, et al. [75]	2015–2016	Determining BF rates and factors associated with BF type and duration in the first year and examine the effect of supplementation, in uncomplicated term births who intended to BF.	Prospective sub-study of RCT	Uncomp term births who intended to BF	(*n* = 655) Sydney, Metropolitan NSW		BF Initiation > 95%	EBF at: 2 months: 81% 6 months: 8% Any BF at: 2 months: 14% 6 months: 84% 12 months: 54% No BF at: 2 months:5% 6 months: 16% 12 months: 46%	
Netting, et al. [76] *Nestle sponsored	2020–2021	Assessing BF rates, duration, use of BM substitutes, introduction of foods, including allergens. The Australian Feeding Infants and Toddler Study 2021 (OzFITS 2021) survey of infant & toddler feeding	Cross-sectional survey	Introduction of formula/solids and food allergens	(*n* = 1,140) Australia-wide (online)		98%	33% had AF in hospital and 70% continued AF after discharge Receiving any AF by age: 40% <1 month olds 51% of 4 month olds 66% of 12 month olds EBF to 6 months < 1% 44% any BF at 12 months 4% any BF at 23 months	**24 months/** **2 years**
Tawia, et al. [77]	2014–2015	Exploring BF practices of ABA volunteers, identifying positive BF practices and modifiable practices, to inform BF promotion.	Cross sectional survey	ABA-trained volunteer BF counsellors	(*n* = 174) Australia-wide (online)			EBF to 6 months 64% BF to 12 months 80% BF at 18 months47.8% BF at 2 years 26.7% BF at 3 years 10.0%	**3 years**
Martin-Kerry, et al. [78]	2011–2013	Assessing dietary data, including beverage consumption and feeding behaviours of young children; and evaluating consistency with Australian dietary guidelines.	Retrospective longitudinal cohort study	Outer-metropolitan (regional) Australians	(*n* = 354) Metropolitan VIC			Any breastmilk: 55.8% at 6 months, 34.3% at 12 months, 5.4% at 24 months, 1.2% at 36 months, 0% at 48 months. Formula: 62% at 6 months, 47% at 12 months, 8.3% at 24 months, 2.4% at 36 months and 0% at 48 months.	**4 years**

ABA – Australian Breastfeeding Association. AF – Artificial Feeding. ATSI – Aboriginal and Torres Strait Islander. BF – Breastfeeding. BFHI – Baby Friendly Health Initiative. BMI- Body Mass Index. CALD – Culturally and Linguistically Diverse. CB- Caesarean Birth. Em CB- Emergency Caesarean birth. EBF – Exclusive Breastfeeding. EBM – Exclusive Breastmilk. EPDS – Edinburgh Postnatal Depression Scale. GDM – Gestational Diabetes Mellitus. LOS – Length of Stay. NB – Norman Birth *we acknowledge this terminology is more accurately reflected as “non-instrumental vaginal birth”. NSW – New South Wales. NT - Northern Territory. NZ – New Zealand. Primps- Primiparous. PN – Postnatal. PPH – Postpartum Haemorrhage. QLD – Queensland. SA – South Australia. T2DM – Type 2 Diabetes Mellitus. Uncomp – Uncomplicated. VIC – Victoria. WA – Western Australia

### Study Characteristics

Of eligible studies, study designs included retrospective or prospective cohort studies (*n* = 22) [17, 40, 42, 43, 46, 48–54, 56, 57, 59, 60, 62, 66, 68, 72, 73, 78], cross-sectional designs (*n* = 12) [39, 44, 45, 47, 55, 58, 64, 65, 67, 70, 76, 77], randomised control trials (RCTs) (*n* = 4) [69, 71, 74, 75], mixed methods (*n* = 2) [61, 63] and one prospective non-randomised intervention trial [41].

From a geographical perspective, most studies involved populations from New South Wales (NSW) (*n* = 12) [17, 46, 49, 51–53, 57–60, 70, 74] and Victoria (VIC) (*n* = 12) [17, 39, 40, 42, 50, 56, 62, 63, 68, 69, 71, 78], followed by Australia-wide (*n* = 8) [47, 55, 61, 65, 67, 72, 76, 77], Queensland (QLD) (*n* = 4) [41, 45, 64, 73], Northern Territory (NT) (*n* = 3) [43, 45, 47], South Australia (SA) (*n* = 2) [44, 54] and Western Australia (WA) (*n* = 2) [45, 66]. There were no eligible studies from Tasmania or the Australian Capital Territory (ACT).

Using the AIHW’s Rural, Remote and Metropolitan Area (RRMA) classifications related to health care access across geographic regions, metropolitan areas are major cities and areas within 20 km distance of a town with a population of 50,000 or more people [79]. Just over half of the studies (*n* = 23) were conducted in metropolitan areas [39, 41, 42, 49–54, 56–59, 61, 62, 66, 68–70, 73–75, 78], with 3 studies in rural areas [46, 48, 60] and 3 reporting from remote areas [43, 44, 47] and the remainder were state-wide or Australia-wide.

Nine studies specifically reported on Indigenous Australian populations [39, 41–48], while seven studies reported specifically CALD Australian populations [49–51, 53–55, 80].

Sample sizes of eligible studies ranged from 40 to 339,854, with three of the included studies reporting on larger samples than the 2010 ANIFS (*n* = 29,000) [17, 40, 51]. The largest (*n* = 339,854) was recruited between 2009 and 2013 [40]. The second largest, from 2018 to 2019, included approximately 200,000 participants across 125 hospitals in NSW and Victoria [40]. The third (*n* = 34,103) was from a Western-Sydney hospital between 2018 and 2021, involving an ethnically diverse population, with 66% of birthing mothers born outside of Australia [51]. The most recent data came from a 2022–2023 study of 1303 mothers [60], a comparable size to annual ABS breastfeeding data, and over half (*n* = 22) of the studies reported sample sizes were less than ABS data.

### Risk of bias within studies

The appropriate JBI Risk of Bias evaluation checklists were used for each study (see supplementary files) with cohort studies receiving high overall quality scores, with some scoring lower for unclear methods for incomplete follow up data. Cross-sectional studies scored lower for not using objective, standard criteria for measuring breastfeeding and study outcomes in a valid and reliable way. Prevalence studies scored lower for poorly reported response rate and management of missing follow up data within the studies. Overall quality and reliability of data within eligible studies was high, while the integration of diverse methods allowed for identifying of population-specific barriers and enablers, in line with the secondary aims of the scoping review.

### Breastfeeding definitions

Almost all of the studies reporting on “exclusive breastfeeding” defined this as receiving only colostrum or breastmilk, whether by directly breastfeeding, or breastmilk provided orally or enterally [67]. Publicly available breastfeeding data for Victoria, Australia, reported “use of any infant formula” was 30.7% and “rate of final feed being taken directly from the breast by breastfed babies” was 72.6% [17]; and only includes babies **≥** 37 weeks gestation [81]. Therefore, breastfeeding exclusivity was derived from those not receiving any formula, at 70.3%. 6/13 Victoria-based studies in this review used state data [17, 40, 42, 50, 56, 62]. NSW state breastfeeding data defined “full breastfeeding” as breastmilk from the breast or expressed for the duration of the admission [17]. Additionally, “partial breastfeeding” and “any breastfeeding” are terms used in NSW data, reflecting infants receiving both breastmilk and formula during admission and at discharge. State-wide data was used in 7 of the 14 NSW studies included [17, 51–53, 58–60]. Studies including preterm infants used the terms: “mother’s own milk”, “any breastmilk” or “no breastmilk”, distinguishing infants receiving pasteurised human donor breastmilk from expressed breastmilk if unable to directly breastfeed [54, 66]. Table 3 summarises definitions and discharge rates across Australian states and territories.

**Table 3:** Breastfeeding reported by Australian State/Territory

	Breastfeeding definitions and annual data	EBF at discharge
**ACT**	Not reported	
**NSW** NSW Mothers and Babies Annual Reporting (2022)	All livebirths discharged home, infant feeding **Full BF**: receiving breastmilk only at hospital discharge **Any BF/Partially BF**: both breastmilk and infant formula at hospital discharge **Formula feeding**: receiving infant formula only at hospital discharge	**70.7%**
**NT**	Not reported	
**QLD** QLD Perinatal Statistics (2022)	**Fluid baby received during the birth episode:** All livebirth discharges receiving the following categories: **BM/colostrum only** **BM/colostrum and infant formula** **Infant formula only** As well as Nil by mouth feeding of each category	**66.9%**
**SA**	Not reported	
**TAS**	Not reported	
**VIC** * VIC Perinatal Services Performance indicators (2022)	Reporting on BF at discharge for live births **≥37 weeks gestation only**. **BF Initiation** **Use of infant formula (any) 30.7%** Rate of final feed taken directly from the breast before discharge **72.6%**	**70.3%** * ≥37 weeks only
**WA**	Not reported	

EBF – Exclusive Breastfeeding. BM –Breastmilk. NSW – New South Wales. NT - Northern Territory. QLD – Queensland. SA – South Australia. VIC – Victoria. WA – Western Australia

### Breastfeeding intention, initiation & duration

Table 2 summarises review findings in order of breastfeeding duration, reflecting most reported breastfeeding up to hospital discharge only, through to longest breastfeeding duration.

### Breastfeeding intention

Overall, ten studies reported on antenatal breastfeeding intention [41, 42, 46, 52, 53, 55, 61, 63, 65, 73], with one reporting breastfeeding intention and initiation but not duration [61]. Breastfeeding intention varied widely, with some diverse cohorts – including Indigenous and CALD Australians – showing over 90% intention to breastfeed [41, 46, 52, 73]. The lowest breastfeeding intentions were 61% in a Chinese Australian cohort [55]; and 77% in an Indigenous Australian group [42], both specifically reporting EBF intention. Lower antenatal breastfeeding intention was associated with reduced rates of exclusive or any breastfeeding [53–55, 65].

### Breastfeeding initiation

Seventeen studies reported on breastfeeding initiation at birth [39, 40, 42, 44, 46, 48, 52, 54–56, 61–63, 65, 73, 75, 76]. Breastfeeding initiation remained above 90% across most studies [40, 42, 55, 75, 76], with lowest rates of initiation at 78.9% seen among mothers with a BMI > 30 [56], and among a number of Indigenous Australian cohorts [44, 46, 48]. Initiation was highest among low-risk, home-birth eligible women who either birthed at home or in hospital at 99% and 98% respectively [62], as well as women in continuity and midwifery-led group practice (MGP) models of care, particularly culturally specific models for Indigenous Australians, compared with standard care models [39, 41, 42, 57].

### Breastfeeding duration

Twelve studies (12/41, 29%) reported on breastfeeding rates at hospital discharge only. In total, over two thirds (28/41, 68%) of studies reported on breastfeeding duration up to 6 months only. Five studies (12%) measured breastfeeding duration up to 12 months while eight studies (19.5%) reported breastfeeding duration extending to 2 years or more. EBF was reported in 29 studies (70.7%). Remaining studies reported breastfeeding rates as any breastfeeding, combining EBF and partial or mixed feeding, compared with no breastfeeding.

EBF at hospital discharge were lowest at 62–64% among CALD mothers, and those with high maternal BMIs [50, 56]. High maternal EPDS (>13), admission to neonatal unit, non-elective caesareans, postpartum haemorrhages, obstetric-led care, maternal diabetes and primiparity were associated with lower EBF rates at discharge [41, 49, 50, 59]. Higher EBF at hospital discharge was among those in midwifery continuity of care models [41, 42, 57, 60, 82]. EBF at discharge was 71% for NSW and 74% for VIC in a large birth cohort (*n* > 200,000), highlighting significantly higher exclusive and any breastfeeding at discharge among BFHI-accredited and public hospitals, using state-based definitions [17].

### Indigenous Australians

Nine studies specifically reported on Indigenous Australian populations and are summarised in Tables 2 and 4. Geographically, eligible Indigenous studies were conducted in greater Melbourne, VIC areas (*n* = 3); regional Tamworth, NSW areas of the Kamilaroi nation (*n* = 2); remote NT communities of an unspecified location (*n* = 1); South Brisbane, QLD (*n* = 1); state-wide SA (*n* = 1); and one study included Indigenous infants from unspecified remote WA, QLD and NSW regions [45]. No studies reported on Indigenous Australian populations in the ACT, TAS or WA alone. Participant language groups and traditional lands of origin were not reported on. One study included Aboriginal infants only [47], while no studies reported only Torres Strait Islander infants. Seven studies recorded breastfeeding duration > 6 months [39, 43–48], while five measured breastfeeding duration of 12 months or longer and the longest duration was up to 5 years [48].

**Table 4:** Summary of breastfeeding barriers and enablers, categorised by population sub-groups

Authors	Study Period	Population Sub-groups	Sample size & Location (city, region/state)	Barriers	Enablers
**INDIGENOUS AUSTRALIANS**					
Ashman, et al. [48]	2012–2016	Indigenous mothers & children	(*n* = 73) Tamworth region, Rural NSW	High maternal **BMI (>30)** Financial constraints Health literacy Busy lifestyles Lack of access to nutritious foods Cultural and family commitments	
Austin, et al. [39]	2009–2014	Aboriginal families	(*n* = 146) Glenelg Shire, Metropolitan VIC		MCH **culturally specific care** model
Brown, et al. [44]	2011–2013	Aboriginal Infants. Culturally specific Models of care.	(*n* = 344) State-wide, SA	Cannabis use in pregnancy **Neonatal unit admission** **Birth weight < 2500 g**	Tertiary **educated** Living outside urban centres Higher maternal stress & social health issues during pregnancy
Kildea, et al. [41]	2013–2019	First Nations infants in BiOC vs standard care models	(*n* = 1,422) Brisbane, Metropolitan QLD	Prematurity (<37 weeks) **Neonatal unit admission** **Birth weight < 2500 g**	BiOC, **culturally specific care** model Unmedicated labour
Leonard, D., et al.	2010–2012	Aboriginal and Torres Strait Islander infants/children aged 6–24 months	(*n* = 227) northern WA, NT, Remote QLD northern WA, NT, Remote QLD	**Non-pay weeks** (income) Dry season	House crowding
Longmore, et al. [43]	2011–2017	Mothers with GDM & T2DM including Indigenous & non-Indigenous populations	(*n* = 1,050) Remote NT	T2DM **C/S birth** **Neonatal unit admission** Maternal obesity **(BMI > 30)**	**Indigenous status** **Remote residence** Gestation > 37 weeks
Onifade, O. M. et al.	2010–2018	Aboriginal and Torres Strait Islander infants	(*n* = 71–246) Tamworth, Rural NSW	BF difficulties (35.8% reported) **Multiparity** Previously non-BF **Neonatal unit admission** Culture & history (colonisation, discrimination)	**Culturally specific care (**First Nations Lactation Consultant)
Springall, T. et al.	2017–2020	Mothers birthing in First Nations continuity of care	(*n* = 211) 3 sites for Baggarrook Yurrongi study in Melbourne, Metropolitan VIC		**Partner/family support** **Spontaneous vaginal birth**
Tonkin, et al. [47]	2017–2018	Aboriginal mothers & children	(*n* = 40) Aboriginal communities, Remote NT	**Pay-cycle weeks** (BF more in non-pay cycle weeks to supplement food).	**Co-sleeping** Children BF on demand at night
**CALD AUSTRALIANS**					
Dahlen, et al. [49]	2012–2013	Australian born vs non-Australian born mothers (CALD) EPDS.	(*n* = 3,092) Sydney, Metropolitan NSW	**EPDS > 13** **Domestic violence** Childhood abuse Anxiety and **depression** Low confidence Recent worry/stress Low emotional support	EPDS < 12
De Mare, et al. [50]	2016–2018	CB vs NB. Ethnicity (CALD) and EPDS	(*n* = 5,520) Epping, Metropolitan VIC	Gestation < 38 weeks **Induced labour** **EPDS > 13** **Asian Ethnicity** NB shorter length of stay (LOS) **BMI > 30** Emergency CB **Male infant**	**Caesarean Birth (longer LOS)** **Multiparous** Spontaneous labour **Married**
Keir, et al. [54]	2018–2020	Late preterm infants (34+0–36+6), CALD	(*n* = 270) Metropolitan, SA	AF as first feed Any **AF in hospital** **CALD**	**BF intention** > 6 months
Kuswara, et al. [55]	2018	Chinese Australians (CALD)	(*n* = 289) Australia-wide (online)		**BF intention** Self-efficacy Awareness of BF guidelines & solids recommendations Longer time living in Australia
Melov, et al. [51]	2018–2021	Pre-COVID vs 1st year of COVID. non-Australian born (66%) CALD population	(*n* = 34,103) Western Sydney, Metropolitan NSW	PN < 1 day admission **(short LOS)** **C/S birth** **Prematurity** **Induced labour**	
Ogbo, et al. [52]	2014	CALD, over 40% overseas-born	(*n* = 17,564) Sydney, Metropolitan NSW	**Maternal age < 20** Maternal smoking **Intimate partner violence** Assisted/operative birth (**C/S birth**) Low SES Pre-existing maternal medical conditions Incorrect positioning/attachment **Prematurity** **Low birth weight < 2500 g** Jaundice	**Partner support** **BFHI accreditation**
Ogbo, et al. [53]	2014–2016	CALD mothers	(*n* = 25,407) Sydney, Metropolitan NSW	Low/no BF intention Smoking in pregnancy **High AN EPDS > 13** **Intimate partner violence** **C/S birth** **Maternal age < 20**	**Partner support** High SES
**High BMI**					
Bish, et al. [56]	2010–2017	Obese (BMI > 30), non-obese	(*n* = 10,234) Metropolitan VIC	Maternal age < 25 **Lower SES** **BMI > 30** Maternal smoking Maternal comorbid medical conditions **C/S birth** (non-vaginal birth) **Neonatal unit admission** Indigenous **Multiparous ≥ 4** **Birth weight < 2500 g**	
Holton, et al. [63]	2020–2021	High BMI **(>25)** vs Normal BMI	(*n* = 80) Melbourne, Metropolitan VIC	Paid employment (76% vs 50% normal BMI), Latching difficulties (48% vs 11% normal BMI), Unrealistic BF expectations Poor body image/confidence **C/S birth** More PN pain Dissatisfied with GP & OB BF support	**Multiparity** BMI < 25 Midwife, Child Health Nurse or **partner/family BF support**
Newby, et al. [73]	2010–2012	BMI < 30 vs BMI > 30	(*n* = 462) Brisbane, Metropolitan QLD	Low comfort BF in public (BMI > 30) **Lower SES** Low education levels	
*NEONATAL UNIT ADMISSION/PRETERM					
Jarrett, et al. [58]	2018	Neonatal readmission vs control	(*n* = 251) Sydney, Metropolitan NSW	**Primiparous** (36% BF difficulties vs 21% multiparous) **Birth weight < 2500 g** (50% BF difficulties vs 25% >2500 g)	Established breastmilk supply (3 or 4 days PN) IV fluids in labour
Jones, et al. [59]	2018–2020	Admission to a neonatal unit vs no admission	(*n* = 1,000) Western Sydney, Metropolitan NSW	Maternal **BMI > 30** Maternal medical conditions (HTN, GDM) **C/S birth** **Ethnicity** (Southeast Asian & Middle Eastern) CALD lack of BF privacy/discreet areas in Neonatal unit EBL > 1000 ml No skin to skin at birth	
Perrella, et al. [66]	2015–2016	Pre-term infants receiving any BM on discharge	(*n* = 49) Perth, Metropolitan WA	Low milk supply **AF in hospital**	Early lactation initiation Frequent & effective expression after birth **BFHI accreditation** Access to Hospital grade pump
**MODELS OF CARE AND BIRTHING FACTORS**					
Bond, et al. [75]	2015–2016	Effect of AF in Hospital on “normal” full-term births, who intended to BF	(*n* = 635) Sydney, Metropolitan NSW	**C/S birth** Low milk supply Problems latching Initial BF delayed **AF before 2 months** **Early return to work** Early solid introduction Dummy/pacifier use Attending daycare	**Previous BF** Tertiary **educated**
Cummins, et al. [57]	2018–2021	Perinatal mental health conditions and models of care	(*n* = 3,028) Newcastle, Metropolitan NSW		Midwife Group Care **Spontaneous vaginal birth**
Fan, et al. [68]	2018–2020	At risk of early BF cessation. Intervention vs control groups.	(*n* = 765) Epping, Metropolitan VIC	Sore nipples Frequency of BF Fussy baby Low supply **Neonatal unit admission** **BMI > 30** Maternal **depression** **C/S birth**	
Flood, et al. [40]	2009–2013	PPH vs Severe PPH	(*n* = 339,854) State-wide, VIC	PPH **Private** maternity care Maternal **BMI > 30** **Multiparous > 4** Maternal age > 45 **Unplanned C/S birth** **North-east Asian ethnicity**; lowest BF rates on discharge	
Forster, et al. [69]	2013–2015	Primiparous receiving BF peer support vs control	(*n* = 1,152) multi-centre, Metropolitan VIC		5 or more contacts with peer-support improved BF duration
McLachlan, et al. [71]	2012–2013	usual care, home visit, home visit & drop in (health clinic)	(*n* = 6,916) State-wide VIC	Maternal age < 25 **C/S birth** Gestation < 37 weeks **Australian-born** Health-care card holder	**Non-Australian born**
Meedya, et al. [17]	2018–2019	NSW vs VIC, Public vs Private, BFHI vs non-BFHI	(*n* = >200,000) State-wide NSW & VIC	**Private hospital** **non-BFHI**	**Public Hospital** **BFHI**
Mollart, et al. [60]	2022–2023	MAPS vs MGP care. Regional Australian	(*n* = 1,303) Outer Newcastle, Rural NSW	**BMI > 30** **C/S birth**	Spontaneous labour **Spontaneous vaginal birth** Non-medicated labour MGP care
Perrella, et al. [61]	2022	non-EL C/S vs EL C/S. Primiparous vs multiparous.	(*n* = 961) Perth, Metropolitan WA	**Unplanned C/S birth** **Primiparity** Higher maternal pain Reduced mobility **Private hospital** Birth complications Conflicting BF information	**Partner stay overnight** in private hospitals sidecar bassinets (easy infant access) appropriate midwife staffing to assist **BFHI accreditation** **Partner support/stay overnight** in public hospitals
Sweet, et al. [62]	2009–2019	Public home birth vs Public hospital birth	(*n* = 774) Melbourne, Metropolitan VIC		**Partner/family support** **Spontaneous vaginal birth**
**OTHER**					
Bailey, et al. [67]	2014–2015	ABA Volunteer Trainees	(*n* = 174) Australia-wide (online)	Low confidence BF problems Conflicting advice **Medicated birth** Lack of knowledge	Bed-sharing/co-sleeping Room sharing **Spontaneous vaginal birth** Uncomplicated pregnancy
Cole, et al. [64]	2017	Any BF vs non-BF. Bed sharing vs non bed-sharing. Pacifier use vs no pacifier use	(*n* = 3,321) State-wide, QLD	Maternal age < 25 **Single** Current smoker Smoked during pregnancy **BMI > 30** **Public birth** Lower SES **Male infants** Multiple birth **C/S birth** **Neonatal unit admission** Received **AF in hospital** Indigenous Infants Pacifier/dummy use infant sleeping in separate room to mother	Maternal birth country non-Aus Tertiary educated **More frequent bedsharing** more likely BF
Leow, et al. [70]	2014–2015	Surveys in English (Australia & Singapore) and Vietnamese (Vietnam)	(*n* = 108) South-west Sydney, Metropolitan NSW	High calorie beverage consumption **Ethnicity** – **Vietnamese**	Higher maternal education
Moss, et al. [72]	2016–2017	EBF to 6 months vs non-EBF	(*n* = 5,340) Australia-wide	Maternal age < 25 Live in major cities **Lower education** **Depression**	**Partnered** **BMI < 25** **Non-smoker**
Netting, et al. [76]	2020–2021	Introduction of formula/solids and food allergens	(*n* = 1,140) Australia-wide (online)	Receiving **AF in hospital** Society/culture Non-BFHI hospitals	
Reynolds, et al. [65]	2019–2020	Intention to BF vs actual BF outcomes	(*n* = 536) Australia-wide (online)	**Lower education** Low income (Govt benefit as main income source)	**BF intention** > 6 months No smoking in pregnancy **Culturally specific MOC**
Tawia, et al. [77]	2014–2015	Trained volunteer BF counsellors (ABA)	(*n* = 174) Australia-wide (online)		Knowledge & **BF intention** **Partner & peer support** Birth outcomes Immediate skin-to-skin Positive BF at work attitude Positive BF in public attitude

ABA – Australian Breastfeeding Association, AF – Artificial Feeding, ATSI – Aboriginal and Torres Strait Islander, BF – Breastfeeding, BFHI – Baby Friendly Health Initiative, BMI- Body Mass Index, CA- Corrected Age, CALD – Culturally and Linguistically Diverse, CB- Caesarean Birth, CGA- Corrected Gestational Age, C/S – Caesarean Section, EBF – Exclusive Breastfeeding, EBM – Exclusive Breastmilk, EPDS – Edinburgh Postnatal Depression Scale, GDM – Gestational Diabetes Mellitus, LOS – Length of Stay, NB – Norman Birth *we acknowledge this terminology is more accurately reflected as “non-instrumental vaginal birth”, NSW – New South Wales, NT - Northern Territory, NZ – New Zealand, PN – Postnatal, PPH – Postpartum Haemorrhage, QLD – Queensland, SA – South Australia, SES – Socio economic status, T2DM – Type 2 Diabetes Mellitus, VIC – Victoria, WA – Western Australia

neonatal unit refers to a specialised infant care unit, in which the infant is separated from the mother, due to requiring higher level care (monitoring or treatment)

Indigenous Australian specific barriers identified were multiparity [46] and pay-cycle weeks [45, 47], with more breastfeeding reported in non-pay cycle weeks. Additionally, enablers included Indigenous status [43], house crowding [45], higher levels of maternal stress and social issues [44] living outside urban areas [43, 44], co-sleeping [47] and culturally specific models of maternity care [39, 41, 46].

Across included studies, data quality and reliability were high. Diverse methods enabled identification of Indigenous-specific barriers and enablers, highlighted differences compared with non-Indigenous populations, and supported comparison of study rigor with ABS and AIHW national breastfeeding data.

### CALD Australians

Six studies reported specifically on CALD Australian populations and are summarised in Tables 2 and 4. CALD was mostly defined as mothers born outside of Australia, categorised as non-Australian born or overseas born [49, 50, 52–54], as well as mothers residing in Australia for 10 years or less before birthing in Australia [55].

Non-Australian born mothers had higher EPDS scores and were significantly more likely to experience PND than Australian born mothers, with domestic violence, intimate partner violence, childhood abuse, anxiety and depression, low confidence, recent worry/stress and lack of emotional support creating breastfeeding barriers [49, 52]. Mothers of Asian ethnicity and CALD mothers with preterm, low birth weight, jaundiced infants or infants admitted to the neonatal care unit, experienced further reduced EBF rates and greater formula use in hospital than Australian-born mothers [50, 54, 59]. Specifically, women of north-east Asian ethnicity experienced lowest rates of exclusive or any breastfeeding on discharge [40] while mothers of south-east Asian and Middle Eastern ethnicity experienced greatest breastfeeding barriers related to lack of privacy in neonatal units [54, 59]. Emergency caesarean birth and vaginal birth with a shorter hospital length of stay (LOS), resulted in less EBF and higher rates of neonatal jaundice readmission for CALD infants [50]. Longer hospital LOS was protective of greater breastfeeding exclusivity for CALD mothers and mothers birthing via caesarean section in public hospitals, due to the ability of a support person/partner to stay overnight in hospital; a practice otherwise prohibited in public hospitals [17, 50, 51].

Being Australian-born was found to be protective in some Asian and Middle-Eastern dominant CALD cohorts [53, 70]; however if the mothers’ country of origin experienced greater breastfeeding exclusivity and duration than that of Australia, it was found to be protective of breastfeeding [59, 71]. Vietnamese and Chinese born mothers had the lowest rates of EBF in hospital [54, 55]. However, among Chinese-born mothers, longer duration of residence in Australia, was associated with increased breastfeeding exclusivity and durations [55].

In the Australian context, Asian ethnicity was identified as a potential barrier to breastfeeding; however substantial variation existed across Asian countries and regions. CALD families had distinct cultural support needs compared with non-CALD populations, including longer hospital stays, continuous presence of a support person, greater need for breastfeeding privacy within neonatal units, and more complex psychosocial considerations, underscoring the importance of a culturally safe care (Table 4).

### Breastfeeding barriers and enablers

Most studies reported on, or mentioned, breastfeeding barriers and/or enablers (39/41, 95%). Of the two that did not, the primary aims of these studies were not related to breastfeeding [74, 78]. Factors associated with EBF, non-EBF or breastfeeding cessation at the chosen postnatal time-points, from hospital discharge through to 4 years postnatal (PN), were measured. Table 4 contains a summary of barriers and enablers to breastfeeding reported where these factors were measured in eligible studies.

Five studies examined breastfeeding among mothers with overweight or obesity (BMI > 30). Consistently finding that higher maternal BMI was associated with lower breastfeeding intention, exclusivity and duration [40, 43, 50, 56, 63, 64, 72, 73]. Eight studies compared breastfeeding outcomes across different models of maternity care, showing that midwifery-led models - including Indigenous and culturally specific care, as well as caseload midwifery care with a known midwife were associated with higher breastfeeding exclusivity and longer duration [41, 44, 57, 60, 61, 63, 83]. While identifying as Indigenous person was generally identified as a barrier to breastfeeding, in remote Australian communities it was associated with increased breastfeeding exclusivity and longer breastfeeding duration [43, 47].

Male infants were less likely to be EBF and experienced shorter duration of any breastfeeding [50, 63, 64]. Infant sleep location in close proximity to the mother (room sharing and/or bedsharing) was an enabler of any breastfeeding, together with greater breastfeeding exclusivity and duration [47, 64, 67]. Low maternal confidence in their ability to breastfeed was associated with less EBF, particularly for women with a high BMI [63, 73] and for CALD mothers [49, 67]. Introduction of solid foods earlier than recommended was identified as a factor in reducing breastfeeding exclusivity in the first six months, particularly among CALD populations [55, 65, 69, 70, 74–76].

Birth in a public hospital was associated with higher rates of EBF on discharge [17, 64, 84], but mothers were more likely to stop breastfeeding by eight weeks postpartum [64]. In contrast, birthing in private hospitals or non-BFHI accredited facilities was associated with lower EBF rates and shorter breastfeeding duration [17, 40, 52, 61, 66, 76, 84]. Mothers with higher socio-economic and education levels consistently demonstrated greater breastfeeding exclusivity and longer duration [44, 52, 53, 56, 64, 70, 72, 73, 75]. Private hospital birth was found to be protective of breastfeeding only in cases of planned caesarean birth [84], largely due to factors such as reduced maternal mobility and the ability for partners to stay overnight and assist with breastfeeding [63]. In both studies, it was noted that increased maternal postnatal pain posed a barrier to breastfeeding.

Factors including caesarean birth, birth complications (emergency birth, postpartum haemorrhage), gestational diabetes (GDM), neonatal unit admission, prematurity (<37 weeks), low birth weight (<2500 g), and in-hospital formula use, were consistently associated with lower EBF rates and predicted breastfeeding cessation within 0–6 months [41, 43, 44, 46, 50, 54, 59, 64, 66, 68, 71, 76]. Maternal smoking before and during pregnancy, maternal cannabis use, pre-existing maternal medical conditions and/or pregnancy complications, were also associated with lower rates of exclusive and any breastfeeding at hospital discharge [42, 44, 52, 53, 59]. Consistent with these findings, a large retrospective Australian population-based cohort study identified outside of the original search strategy showed intrapartum interventions were associated with reduced EBF at discharge and up to six months postpartum, with increasing numbers of interventions linked to earlier breastfeeding cessation [85].

Four studies explored the relationship between breastfeeding and mental health [49, 50, 53, 75]. Three used the EPDS to assess depression [49, 50, 53], while one study employed the State-Trait Anxiety Inventory (STAI-6) to examine anxiety in relation to breastfeeding [49]. High antenatal EPDS scores (>13) and a history of maternal depression were associated with lower rates of EBF [68, 72]. Elevated postnatal EPDS and anxiety were frequently associated with early breastfeeding cessation, particularly among CALD mothers [40, 49, 75]. Additionally, intimate partner violence and domestic violence were identified as barriers to breastfeeding in three studies focused on CALD populations [49, 52, 53]. In contrast, among Indigenous Australians, factors including household crowding, higher reported levels of stress, and pay-cycle weeks were unexpectedly associated with increased breastfeeding [42, 44, 45].

Studies reported conflicting findings regarding the influence of maternal age, parity and ethnicity with breastfeeding. Younger mothers (<25 years old) were consistently associated with lower rates of exclusive or any breastfeeding, as well as shorter breastfeeding duration [52, 53, 64, 71, 72]. Older maternal age, >45 years, was also linked to reduced breastfeeding exclusivity and shorter duration [40]. Parity showed mixed associations. Multiparity was identified as both a breastfeeding barrier [40, 46] and enabler [50, 58, 63] depending on context and population. Primiparity, however, was predictive of non-EBF [58, 61]. Single mothers were less likely to EBF and tended to breastfeed for shorter durations [64] compared to partnered or married mothers [50, 53, 62, 63]. Having a supportive partner was protective for breastfeeding, particularly among CALD mothers, and in contexts involving maternal depression and younger maternal age [52, 53, 62, 72, 84].

Overall, BFHI facilities, midwifery-led care, culturally specific care and continuity of care were factors associated with higher breastfeeding exclusivity and duration. CALD Australians, maternal BMI > 30, substance use, younger mothers and poorer maternal psychosocial wellbeing (mental health, personal stress, lack of support, social isolation) consistently experienced poorer breastfeeding outcomes. Additionally, birthing environments in the context of Caesarean birth, birth complications, GDM management, prematurity, low birth weight, neonatal unit admission were consistently associated with poorer breastfeeding outcomes.

## Discussion

This review found that overall rates of any and exclusive breastfeeding in Australia have remained largely unchanged since the 2010 ANIFS, highlighting the urgent need for a comprehensive national review of breastfeeding practices, policies and programs - consistent with the WHO’s recommendation for five-yearly evaluations. Our review found a mothers’ country of origin to be protective of breastfeeding, if the country of origin experienced higher breastfeeding exclusivity and longevity than Australia [59, 71]. This indicates supportive societal influences on infant care practices and national policy that protects women’s and children’s human right to breastfeed could achieve a paradigm shift in breastfeeding practices.

Our review identified inconsistencies in how breastfeeding was defined and measured in national reporting and academic literature, hindering accurate data interpretation. Among states reporting non-mandatory breastfeeding data, conflicting definitions and methods make it difficult to determine exclusivity. This underscores the need for a national consensus to ensure reliable EBF estimates and early breastfeeding cessation rates. Recognising breastfeeding as a key health indicator in Australia is critical – not only to support, protect and promote breastfeeding, but also to address persistent gender inequities and contribute to economic resilience, climate action, and sustainability through coordinated, evidence-based policy.

Together, these findings identify clear system-level gaps and underpin four key recommendations to strengthen surveillance, policy coherence, and equity-focused breastfeeding support in Australia:

## Recommendations

### Establish breastfeeding as a national health indicator through standardised definitions and integrated national data collection

Despite clear health benefits, breastfeeding is not currently recognised as a national health indicator in Australia. The AIHW’s National Core Maternal Health Indicators – mandated under the Australian Health Performance Framework (AHPF) – do not track breastfeeding or infant feeding methods at birth or hospital discharge. This gap limits policy accountability, undermines efforts to improve breastfeeding rates at a systemic level, and constrains progress toward health equity.

The 2019 *National Breastfeeding Strategy* highlighted the need for a national data collection framework to support effective monitoring and intervention [21]. Our review found significant inconsistencies in how breastfeeding is defined and measured across national reporting and academic literature, complicating accurate interpretation of trends. Establishing a national consensus on definitions, coupled with consistent data collection, would allow for more precise tracking of breastfeeding prevalence, exclusivity, and duration.

Integrating breastfeeding data collection into existing national systems – such as child health records or the immunisation schedule, offers a practical solution. The Victorian Child Development Information System (CDIS) collects ongoing breastfeeding data from 0 to 5 years through the state-wide Maternal and Child Health (MCH) service, achieving retention of 85.8% at 8-months; This model provides an exemplar framework for potential national implementation [86].

Without comprehensive data, Australia cannot effectively monitor progress against health targets, allocate resources or address disparities. This is particularly critical for improving long term health outcomes among marginalised CALD and Indigenous populations. Recognising breastfeeding as a national health indicator is essential for achieving gender and health equity improving maternal and child health outcomes, supporting the national “Closing the Gap 2032” strategy [87, 88].

### Strengthening breastfeeding support: the role of BFHI and protection from infant formula marketing

Most births in Australia occur in hospitals, and BFHI accreditation is associated with significantly increased EBF rates [2]. Yet limited BFHI accreditation in Australian hospitals remains a major health system barrier [17, 18], underscoring the urgent need to expand BFHI nationally, to protect, promote and support breastfeeding [20, 89]. BFHI initiatives ensure ongoing staff education and training, enabling skilled support for diverse mother-infant dyads and addressing persistent structural barriers in birthing environments.

Culturally safe BFHI practices in Australian public hospitals, such as allowing a support person/partner to stay overnight to assist with baby cares during rooming-in, support breastfeeding.

Beyond BFHI, stronger regulation is needed to protect infant feeding from unethical formula marketing. International experience demonstrates the impact of policy: in Malawi, Cambodia and particularly Sri Lanka, mandatory legislation restricting infant formula marketing was associated with increased EBF rates – for infants 0–6 months, from just 17% to 76% over a 12 year period [2]. In Australia, Commonwealth oversight remains limited, with free or discounted formula distributed to hospitals, compromising commercial-free birthing environments.

Together, expanded BFHI coverage and robust legislative protections provide evidence-based strategies to increase breastfeeding rates, reduce inequities, and safeguard long-term maternal and child health.

### Strengthen parental leave and workplace breastfeeding protections

Globally, the return to paid employment is the frequently reported reason for early cessation of breastfeeding and failure to achieve EBF goals [19, 24, 90, 91]. WHO data show a large global drop in EBF rates between 3 and 5 months of age, with no country exceeding 60% EBF beyond 4 months [92]. In Australia, this trend is mirrored by a significant decline in exclusive and any breastfeeding between 3 and 5 months postpartum; aligning closely with the 20-week government paid parental leave period [7, 32]. These findings suggest that current parental leave and workplace conditions are insufficient to support families to sustain optimal breastfeeding and should be improved to elevate breastfeeding as a national public health investment, bridging persistent health disparities for marginalised populations.

Mandated workplace protections, such as paid lactation breaks, designated spaces with clean facilities, and flexible work arrangements, are essential to support continued breastfeeding [23] and are protected in Australia under the *Sex Discrimination Act 1984* [26]. These are recognised not only as public health measures but as basic human rights of every mother and infant [19]. A recent systematic review confirmed that countries with mandated workplace breastfeeding legislation achieved longer breastfeeding durations and higher exclusivity rates after mothers return to paid employment [93]. In contrast, Australia’s voluntary workplace protections leave breastfeeding support subject to individual negotiation with employers [94, 95]. The global and national decline in breastfeeding following return to work highlights the urgent need for stronger parental leave and workplace legislation, empowering women to meet their breastfeeding goals.

### Recognise breastmilk in national economic and sustainability frameworks

Breastmilk is a renewable, environmentally sustainable food source with a low-carbon footprint, offering unmatched public health benefits while contributing to national food security and economic resilience [96–98]. Despite its value, breastmilk remains largely invisible in economic, climate, energy and food system policies. Australian policy makers should integrate breastfeeding and breastmilk production into economic modelling, climate targets and food system planning – to help address gender and health inequities created by decades of policy neglect [89]. Recognising breastmilk’s contribution in GDP calculations [14], as recommended by the United Nations and adopted in countries including Norway, provides a mechanism to quantify its societal value [15, 25]. Including breastmilk within national sustainability and food security frameworks would support cohesive, future proof public health, economic, and environmental policy. Acknowledging its role in national emergency and disaster preparedness further strengthens system resilience by protecting infant feeding during crises [23, 99].

The AIHW 2023 breastfeeding report also highlights the environmental burden of artificial formula feeding, which produces twice the carbon footprint of breastfeeding due to manufacturing emissions [7]. Achieving global nutrition targets through optimal infant breastfeeding practices would therefore yield greater environmental benefit than decarbonising the formula manufacturing industry alone, while supporting breastmilk as a high quality, local, and sustainable food source [96–98]. Embedding principles of justice and non-maleficence within economic and climate policy supports investments in breastfeeding, reducing reliance on artificial milks and their associated environmental and economic costs [96, 97]. Elevating breastmilk within policy frameworks is therefore not only an investment in maternal and child health, but in Australia’s economic and climate future.

## Strengths/limitations

To our knowledge, this is the first scoping review of national EBF rates in contemporary Australian populations since the 2010 ANIFS, providing timely data relating to 2025 national breastfeeding targets, with implications on Closing the Gap 2032 targets. The methodology adopted allowed for rigorous interpretation of diverse data, identification of gaps in national breastfeeding data collection methods, definitions, outcome measures and social and structural supports within studies of Australian populations. However, since the scoping review does not have a formal methodological evaluation framework to critique quality, the interpretation of evidence is limited. Conflicting definitions of exclusive breastfeeding within the literature made it difficult to accurately determine exclusivity, a limitation of the included studies that highlighted a national consensus is necessary.

A limited grey literature search could have excluded relevant studies. Additionally, no studies reported solely on Torres Strait Islander populations; acknowledging the culturally unique child-rearing practices, including breastfeeding, may not be accurately reflected by Indigenous Australian populations within this review. The authors wish to acknowledge Indigenous data sovereignty and the limitations of secondary data presented within this paper for First Nations Australian populations.

## Conclusion

Recognising breastfeeding as the foundation for global human health and survival, begins with national commitment to ongoing protection, promotion and support of breastfeeding within climate and economic policy [1]. Inconsistencies in breastfeeding definitions, and data collection methods result in unreliable breastfeeding monitoring. Establishing nationally cohesive collection methods, would better inform investment into targeted support strategies, addressing long term health disparities of marginalised Australian communities [20, 100]. Barriers to achieving optimal breastfeeding can be overcome by recognising breastmilk and breastfeeding as such, vital to supporting sustainable health, workplace, economic, climate and energy systems [25].

## Electronic supplementary material

Below is the link to the electronic supplementary material.

Supplementary Material 1

## Data Availability

Not applicable.
