# Supplementary material for: Breastfeeding among contemporary Australian populations and 2025 national targets: a scoping review of current data and implications for policy and practice
Source: Int Breastfeed J. 2026 Apr 14;21:53. doi: 10.1186/s13006-026-00826-9 (PMC13227678; doi:10.1186/s13006-026-00826-9)
Supplement: Supplementary file 1 — Supplementary Material 1 [file 13006_2026_826_MOESM1_ESM.docx]

**Supplementary Materials:**

1. **JBI Risk of Bias for articles included in the scoping review**

**JBI Risk of Bias: Cohort Studies: n=22**

Q1. Were the two groups similar and recruited from the same population?
Q2. Were the exposures measured similarly to assign people to both exposed and unexposed groups?
Q3. Was the exposure measured in a valid and reliable way?
Q4. Were confounding factors identified?
Q5. Were strategies to deal with confounding factors stated?
Q6. Were the groups/participants free of the outcome at the start of the study (or at the moment of exposure)?
Q7. Were the outcomes measured in a valid and reliable way?
Q8. Was the follow up time reported and sufficient to be long enough for outcomes to occur?
Q9. Was follow up complete, and if not, were the reasons to loss to follow up described and explored?
Q10. Were strategies to address incomplete follow up utilized?
Q11. Was appropriate statistical analysis used?

all questions answered with: **Y (yes) N (no) U (unclear) N/A (not applicable)** Overall appraisal: (I) Include (E) Exclude (S) Seek further info

|  | **Q1** | **Q2** | **Q3** | **Q4** | **Q5** | **Q6** | **Q7** | **Q8** | **Q9** | **Q10** | **Q11** | **Overall** |
| --- | --- | --- | --- | --- | --- | --- | --- | --- | --- | --- | --- | --- |
| Ashman, A. M.; et al. | Y | Y | Y | Y | Y | Y | Y | Y | Y | Y | Y | I |
| Bish, M. R.; et al. | Y | Y | Y | Y | Y | Y | Y | Y | Y | N/A | Y | I |
| Cummins, A.; et al. | Y | Y | Y | Y | N | Y | Y | Y | Y | Y | Y | I |
| Dahlen, H. G.; et al. | Y | Y | Y | Y | N | Y | Y | Y | Y | Y | Y | I |
| De Mare, K. E.; et al. | Y | Y | Y | Y | Y | Y | Y | Y | Y | N/A | Y | I |
| Fan, W. Q.; et al. | Y | Y | Y | Y | Y | Y | Y | Y | Y | N | Y | I |
| Flood, M. M.; et al. | Y | Y | Y | Y | Y | Y | Y | Y | Y | N/A | Y | I |
| Jones, R. A.; et al. | Y | Y | Y | Y | Y | Y | Y | Y | Y | N/A | Y | I |
| Keir, A.; et al. | Y | Y | Y | Y | Y | Y | Y | Y | Y | Y | Y | I |
| Longmore, D. K.; et al. | Y | Y | Y | Y | U | Y | Y | Y | Y | U | Y | I |
| Martin-Kerry, J.; et al. | U | Y | Y | Y | N | Y | Y | Y | Y | U | Y | I |
| Meedya, et al. | Y | Y | Y | Y | Y | Y | Y | Y | Y | N/A | Y | I |
| Melov, S. J.; et al. | Y | Y | Y | Y | Y | Y | Y | U | Y | Y | Y | I |
| Mollart, L.; et al. | Y | Y | Y | Y | N | Y | Y | Y | Y | N/A | Y | I |
| Moss, K. M.; et al. | U | Y | Y | Y | Y | Y | Y | Y | Y | U | Y | I |
| Newby, R. M.; et al. | Y | Y | Y | Y | Y | Y | Y | Y | Y | Y | Y | I |
| Ogbo, F. A.; et al. | Y | Y | Y | Y | Y | Y | Y | Y | Y | N/A | Y | I |
| Ogbo, F. A.; et al. | Y | Y | Y | Y | Y | Y | Y | Y | Y | N/A | Y | I |
| Onifade, O. M.; et al. | Y | Y | Y | Y | Y | Y | Y | Y | Y | Y | Y | I |
| Perrella, S. L.; et al. | Y | Y | Y | Y | Y | Y | Y | Y | Y | U | U | I |
| Springall, T l; et al. | Y | Y | Y | Y | Y | Y | Y | Y | Y | N/A | Y | I |
| Sweet, L; et al. | Y | Y | Y | Y | Y | Y | Y | Y | Y | N/A | Y | I |

**JBI Risk of Bias:** **Cross Sectional Studies: n=12**

Moola S, Munn Z, Tufanaru C, Aromataris E, Sears K, Sfetcu R, Currie M, Qureshi R, Mattis P, Lisy K, Mu P-F. Chapter 7: Systematic reviews of etiology and risk . In: Aromataris E, Munn Z (Editors). JBI Manual for Evidence Synthesis. JBI, 2020.

Q1. Were the criteria for inclusion in the sample clearly defined?
Q2. Were the study subjects and the setting described in detail?
Q3. Was the exposure measured in a valid and reliable way?
Q4. Were objective, standard criteria used for measurement of the condition?
Q5. Were confounding factors identified?
Q6. Were strategies to deal with confounding factors stated?
Q7. Were the outcomes measured in a valid and reliable way?
Q8. Was appropriate statistical analysis used?

all questions answered with: **Y (yes) / N (no) / U (unclear) / N/A (not applicable)** Overall appraisal: (I) Include (E) Exclude (S) Seek further info

| **Author/s** | **Q1** | **Q2** | **Q3** | **Q4** | **Q5** | **Q6** | **Q7** | **Q8** | **Overall** |
| --- | --- | --- | --- | --- | --- | --- | --- | --- | --- |
| Austin, C.; Arabena, K. | Y | Y | Y | Y | N | Y | U | Y | I |
| Bailey, C; at al. | Y | N | N | N | U | N | N | U | I |
| Brown, S.; et al. | Y | Y | Y | Y | Y | Y | Y | Y | I |
| Cole, R., et al. | Y | Y | Y | Y | Y | Y | Y | Y | I |
| Jarrett, O.; et al | Y | Y | Y | Y | Y | Y | U | Y | I |
| Kuswara, K.; et al | Y | Y | Y | Y | U | Y | U | Y | I |
| Leonard, D.; et al | Y | Y | U | Y | Y | U | Y | Y | I |
| Leow, T. Y. Q.; et al. | U | U | Y | Y | Y | U | Y | Y | I |
| Netting, M. J.; et al. ***Nestle funded** | U | Y | Y | U | Y | Y | Y | Y | I |
| Reynolds, R.; et al. | Y | Y | Y | U | Y | N | Y | U | I |
| Tawia, S.; et al. | Y | U | Y | U | Y | U | Y | Y | I |
| Tonkin, E.; et al. | Y | Y | Y | Y | Y | Y | Y | Y | I |

**JBI Risk of Bias: Prevalence studies: n=7**

Munn Z, Moola S, Lisy K, Riitano D, Tufanaru C. Chapter 5: Systematic reviews of prevalence and incidence. In: Aromataris E, Munn Z (Editors). JBI Manual for Evidence Synthesis. JBI, 2020

1. Was the sample frame appropriate to address the target population?
2. Were study participants sampled in an appropriate way?
3. Was the sample size adequate?
4. Were the study subjects and the setting described in detail?
5. Was the data analysis conducted with sufficient coverage of the identified sample?
6. Were valid methods used for the identification of the condition?
7. Was the condition measured in a standard, reliable way for all participants?
8. Was there appropriate statistical analysis?
9. Was the response rate adequate, and if not, was the low response rate managed appropriately?

all questions answered with: **Y (yes) / N (no) / U (unclear) / N/A (not applicable)** Overall appraisal: (I) Include (E) Exclude (S) Seek further info

| **Author/s** | **Q1** | **Q2** | **Q3** | **Q4** | **Q5** | **Q6** | **Q7** | **Q8** | **Q9** | **Overall** |
| --- | --- | --- | --- | --- | --- | --- | --- | --- | --- | --- |
| Bond D. M.; et al | U | Y | Y | Y | Y | Y | Y | Y | U | I |
| Forster, D. A.; et al. | Y | Y | Y | Y | Y | Y | Y | Y | Y | I |
| Holton, S.; et al. | Y | Y | N | Y | Y | Y | Y | Y | U | I |
| Kildea S.; et al. | Y | Y | Y | Y | Y | Y | Y | Y | Y | I |
| McLachlan, H. L.; et al. | Y | Y | Y | Y | Y | Y | Y | Y | Y | I |
| Perrella, S. L.; et al. | Y | Y | Y | Y | Y | Y | Y | Y | U | I |
| Wen, L. M.; et al. | Y | Y | Y | U | Y | Y | Y | Y | U | I |

1. **Data Extraction Template used for scoping RV:**

| **Author/s** | **Year** | **Title** | **Design/Method** | **Location** | **Sample size** | **Study period** | **Study aim/s** | **BF measures** | **Key Findings** | **Population sub-groups** | **Barriers and/or Enablers** | **Journal** | **Link** |
| --- | --- | --- | --- | --- | --- | --- | --- | --- | --- | --- | --- | --- | --- |

1. **Search Terms for databases**

Studies were identified from relevant databases: CINAHL, Medline, PubMed, Scopus, Cochrane and Embase in April 2025, using identified keywords and index terms. Breastfeeding search terms used: “exclusive breastfeeding” “full breastfeeding” “partial breastfeeding” “combination feeding” “mixed feeding” “any breastfeeding” “predominant breastfeeding: “no breastfeeding”, “non-exclusive breastfeeding” or reported as receiving “mothers own milk” or “any breastmilk” or “no breastmilk”.

The search terms: infant feeding or breastfeeding or breastfeeding or bottle feeding or formula feeding AND barriers or obstacles or challenges or difficulties or issues or problems AND enablers or facilitators or factors or support AND prevalence or duration or incidence. The Geographic subset criteria entered was “Australia or Australasia or Oceania” if geographic classification of Australia was available on the database. Additionally, the words were separated (eg: “breast milk” “breast feeding” “human milk”), in subsequent searches of grey literature, to further capture terms used in the literature.
